# Predicting anxiety-related personality traits by means of serotonergic VNTR variants STin2 and 5-HTTLPR

**DOI:** 10.1016/j.xjmad.2023.100031

**Published:** 2023-10-07

**Authors:** Andrea Felten, Thomas Plieger, Martin Reuter

**Affiliations:** Department of Personality and Biological Psychology, Laboratory of Neurogenetics, University of Bonn, Bonn, Germany

**Keywords:** SLC6A4 polymorphisms, 5-HTTLPR, STin2 VNTR, Rs25531, Neuroticism, Harm avoidance

## Abstract

Polymorphisms of the serotonergic system are amongst the most commonly investigated genetic variants with respect to anxiety-related personality traits and affective disorders. Mostly the prominent 5-HTTLPR, a functional VNTR in the 5-HTT promoter region, is intensively analysed but effect sizes in meta-analyses are small and results are inconsistent. We reinvestigated the association of 5-HTTLPR with harm avoidance (HA) and neuroticism taking another functional 5-HTT-VNTR (STin2) into account, as both VNTRs have transcription regulating properties and research points to combinatorial effects on transcription efficacy. N = 2969 participants, among them 447 inpatients suffering from affective disorders, were genotyped and filled in the TCI, NEO-FFI personality inventories besides the CLEq measuring the extent of experienced stressful life events. Significant main effects for the 5-HTTLPR with inpatients carrying the L+ allele having lower HA scores as well as for the STin2 with healthy controls carrying at least one STin2.12R allele having lower neuroticism scores were observed. Besides no gene-interaction occurred. However, specific haplotype effects were observed in healthy participants as well as in the total sample. More specifically, the 12/L variant was associated with significant lower HA and neuroticism scores. Results highlight the multifactorial interplay of 5-HTT genetic variants and the use of haplotypes in association studies on anxiety-related personality traits with impact on affective disorders.

## Introduction

Individual differences in personality depend on environmental and genetic factors, with the latter accounting for approximately 30–40% of the variance in personality traits [1]. Assessing individual differences in regard to negative emotionality by molecular genetics and functional neuroimaging in the last twenty-five years has pointed to some of the underlying mechanisms but we are still in the very beginning of understanding the molecular genetic basis of negative emotionality-related traits.

Cloninger’s psychobiological model of personality [2] provides a link between specific temperamental traits and neurotransmitter systems, with Harm Avoidance (HA) being predominantly linked to the serotonergic (5-HT) system. Accordingly, there are multiple reported associations between components of the serotonergic system and personality traits of negative emotionality, like Harm Avoidance or neuroticism [3] as well as with affective disorders, connecting the serotonergic system with a vulnerability to negative affect in the normal and pathological range.

The serotonin transporter (5-HTT) is a key component of serotonergic neurotransmission playing an important role in serotonin homeostasis and bioavailability by regulating 5-HT reuptake into the pre-synapse [4]. Accordingly, 5-HTT is the major target of the selective serotonin reuptake inhibitors [5] that competitively block substrate binding and thereby prolong neurotransmitter action at the synapse. Gene variants influencing 5-HTT functioning, especially 5-HT reuptake efficiency, as much as 40-fold in vitro [6] contribute to individual differences in the efficiency of SSRI treatment as well as anxiety-related personality traits [7].

One of the most extensively investigated genetic variants in the context of negative emotionality and affective disorders is the 5-HTT gene-linked polymorphic region (5-HTTLPR). The 5-HTTLPR constitutes a variable number of tandem repeat (VNTR) polymorphism located in the proximal promoter region 1.4 kb upstream of the transcription start site of the *SLC6A4* gene. The VNTR composition shows 40 different allelic variants currently known [8] with the insertion or deletion of 20–24 bp repeat units leading to the most common alleles: the long (L) or short (S) allele. As reviewed by Iurescia et al. [8], despite some controversial results [9], [10], the majority of in vitro expression studies largely confirmed higher transcriptional activity of the promoter containing the L-allele [11], [12], [13]. Furthermore, the mRNA transcription efficacy is modulated by the single nucleotide polymorphism (SNP) rs25531, an A to G substitution located within the 5-HTTLPR. The G allele leads to reduced expression [13] and because the S_G_ allele frequency is lower than 1% in humans [14] as well as it is almost in phase with the L allele [15], the HTTLPR is often considered a triallelic polymorphism with L_A_ allele (L’) as the highest expressing and L_G_ and S_A_ alleles (S’) as the low expressing haplotypes.

The serotonin transporter intron 2 (STin2) VNTR comprises three common allelic variants with nine (STin2.9), ten (STin2.10), or twelve (STin2.12) copies of a 16- or 17 bp repeat [16]. Different repeat number within the VNTR supports differential expression in vitro [17], [18] and both differential and tissue-specific expression in a transgenic model [19], with STin2.12 and STin2.9 having stronger enhancer-like properties than the 10-repeat allele has.

Both functional VNTR polymorphisms have been associated with a range of behavioural and psychiatric disorders including depression, schizophrenia, obsessive-compulsive disorder, and anxiety [20]. Besides, associations with personality traits are reported whereby the S allele of 5-HTTLPR seems to be associated with higher neuroticism and HA scores overall, although gene association studies as well as meta-analyses report inconsistent findings with only small effects and albeit weak association for both dimensions of negative emotionality [21], [22]. Inclusion of the rs25531 still revealed mixed results, but again with a tendency towards higher levels of negative emotionality in S’ allele carriers [23]. Similarly, for the STin2 VNTR the strength and nature of any association is still uncertain [24]. Of course, a lot of confounding factors, mainly sample size, population heterogeneity in terms of sex, age, educational or cultural background, and use of different personality inventories, exist as discussed in the meta-analyses. Moreover, gene-environment interactions influencing 5-HTT expression by epigenetic regulation as well as additional functional genetic variants in *SLC6A4* gene further modulate 5-HTT functionality and expression with implications for anxiety-related disorders and personality traits [25], [26], [27]. Overall, it is surprising that studies investigating both VNTRs simultaneously regarding personality traits are scarce, despite the given functionality of both VNTRs. However, Genome-wide association studies currently evidence that differences in genetic variation that change gene expression, rather than variation that changes protein-coding sequences, largely contribute to the risk for complex genetic traits [28], [29]. Genetic variation in non-coding regulatory regions often interacts via long-distance interactions thereby differentially influencing transcriptional responses to developmental signals as well as environmental stressors which could shape our wellbeing and mental health [30]. Accordingly, reporter-gene studies using dual reporter gene constructs, where both VNTRs can be analysed together with regard to their transcriptional efficiency, revealed combinatorial effects of both VNTRs on gene expression [31]. In the study of Ali and colleagues, the combinatorial variation could not be predicted from the individual VNTR activity demonstrating that both VNTRs act in concert. Regarding personality traits HA and neuroticism, only few studies consider both VNTRs. Saiz and colleagues showed that the L12 haplotype was associated with lower HA in n = 400 healthy Spanish Caucasians [32]. By contrast, in a sample of n = 301 healthy Russian participants’ individuals with S12 haplotype scored higher on HA whereas the opposite association was observed between S10 and HA. Moreover, it was reported that the presence of STin2.12 predisposes to higher HA scores [3]. However, another study with n = 204 elderly people over 60 years found no association between STin2/5-HTTLPR haplotypes and neuroticism [33].

To sum up, there is an extensive and constantly growing literature on the 5-HTTLPR and STin2 genetic variants but findings with respect to negative emotionality are still controversial. Only few studies to date examined potential additive or genetic interaction effects of these functional variants on personality traits HA and neuroticism. In addition, sample sizes often lack power. Moreover, the potentially modifying effects of the SNP rs25531, and gene-environment interactions such as stressful life events have mostly been neglected. Our aim was to reinvestigate, whether allelic combinations of both VNTRs while considering rs25531 lead to a better understanding of 5-HT-related individual differences in HA and neuroticism in a representative sample of healthy Caucasians as well as inpatients suffering from burnout/stress-related psychopathology. Although we assume that there is the same biological continuum underlying phenotypic variability in the normal and psychopathological range, it is worth investigating if the same findings hold in groups of healthy participants and people suffering from affective disorders.

## Methods and materials

### 2.1 Participants

Overall, our sample comprises n = 2969 participants (n = 967 men and n = 2002 women, age: M= 34.30 years, S.D.: 13.49, range: 18–77 years). N = 1345 are members of the Bonn Gene Brain Behaviour Project (BGBBP) and n = 1624 represent participants of a broad project measuring the biological basis of depression and burnout. The latter sample has already been described in more detail in former studies [34], [35] and comprises n = 1177 German employees from a wide range of professions and n = 447 inpatients, recruited in psychosomatic clinics, suffering from burnout and/or depression. BGBBP members are mainly psychology students. The three samples are named “students” (younger, healthy participants mainly students), “middle-aged” (older, healthy participants mainly employed), and “inpatients” (older participants suffering from burnout/depression) in the following. All participants have given written consent to participate and to use of their data for molecular genetic research, which was approved by the local ethics committee at the University of Bonn. Because 5-HTT allele frequencies depend on ethnicity [15] only Caucasians were included.

### Genotyping

Automated purification of genomic DNA was conducted by means of the MagNA Pure 96 LC System using a commercial extraction kit (MagNA Pure 96 DNA and Viral NA Small Volume Kit; Roche Diagnostics, Germany). 5-HTTLPR and rs25531 genotyping was performed as previously described [36] with slight modifications. In brief, after an initial denaturation for 6 min at 94 °C, 37 cycles of denaturing at 94 °C for 30 s, annealing at 64 °C for 1.5 min, and extension at 72 °C for 1 min were followed by a final extension at 72 °C for 5 min. PCR amplification was carried out in a final volume of 25 µl consisting of 25 ng DNA, 0.5 mM dNTPs, 0.25 μM of forward 5′-TCCTCCGCTTTGGCGCCTCTTCC-3′ and reverse 5′-TGGGGGTTGCAGGGGAGATCCTG-3′ primers, 1 mM MgCl_2_, 5.3% DMSO, and 0.5 U of Diamond Taq polymerase (Eurogentec, Germany). To genotype rs25531 PCR products were digested with MSP1 (New England Biolabs) and incubated at 37 °C for about 1 h 15 min and afterwards samples were analyzed by 1.7% agarose gel electrophoresis. Two participants with rare extra-large L-alleles were excluded from further analyses due to very low frequency as well as inconclusive transcriptional effects of this variant.

STin2 genotyping was performed according to [37] with some modifications. STin2 VNTR region was amplified by polymerase chain reaction in a final volume of 12 µl, using 15 ng of DNA, 1.5 mM dNTPs, 0.15 µM of each primer 5´-GTCAGTATCACAGGCTGCGAG-3´ (forward primer), 5´-TGTTCCTAGTCTTACGCCAGTG-3´ (reverse primer) and 1 U of Diamond Taq polymerase. The initial denaturation step of three minutes at 95 °C was followed by 36 cycles each of 95 °C for 45 s, 59 °C for 45 s and 72 °C for 1 min. PCR was terminated by 72 °C for 4 min 10 µl of resulting PCR product was separated on a 2% agarose gel by electrophoresis. Amplification product sizes were 302 bp (12 repeats), 268 bp (10 repeats) or 251 bp (9 repeats), respectively.

### Measurements and statistical analyses

All participants completed the German version of Cloninger’s Temperament and Character Inventory to measure HA. To assess neuroticism, the student subsample filled in the NEO Five Factor Inventory (NEO-FFI) whereas the middle-aged and inpatients answered the Big Five Inventory-10 (BFI-10) [38]. Therefore, the mean values of neuroticism were analysed separately in the subsamples and correlated with HA in the first step. The subscales HA and neuroticism were highly correlated independent of neuroticism measure (student sample measured by NEO-FFI: r = .663, p < .001; middle-aged and inpatients measured by BFI-10: r = .671, p < .001). In the next step, we checked whether the correlations between HA and neuroticism were different dependent on neuroticism measure (i.e. NEO-FFI or BFI-10 in the different samples), which was not the case (z = 0.39, p = .696). Therefore, we decided to treat the neuroticism mean scores for both measures as equal and calculated the association between HA and neuroticism for the entire sample (r = .654, p < .001).

For most participants (N = 2461) data of the CLEq (critical life events questionnaire, a self-constructed questionnaire) measuring the extent of experienced stressful life events (SLEs) were available. The self-constructed questionnaire is described in more detail in [34], [39]. The influence of the control variables age and SLEs on variables of interest was tested by means of Pearson product-moment correlations (two-tailed). SLEs x 5-HTTLPR genotype interaction was analysed by ANCOVA with SLEs dichotomized by median split.

To investigate effects of polymorphic loci on HA and neuroticism two-way univariate analyses of variance were performed. Analyses were conducted with the following independent factors: 5-HTTLPR and triallelic genotype, biallelic dominant S model (S+: S/S + S/L versus L/L) and L model (L+: L/L + L/S versus S/S) as well as triallelic dominant S (triallelicS+: S/S + S/LA + S/LG + LG/LG + LA/LG versus LA/LA) and L model (triallelicL+: LA/LA + LA/LG + LA/SA + LA/S versus S/S + LG/LG + LG/S). Because STin2.9 allele frequencies are rather low (2.7%), analyses were performed on the allelic level with STin2 alleles grouped against 10R (10/10, 10/12, 10/9 versus 12/12, 12/9) or 12R (12/12, 12/10, 12/9 versus 10/10, 10/9), respectively. Note that the rare STin2.9 allele did not change the results whether it is included in the analyses or not.

PHASE version 2.1 [40], [41] was used to infer haplotype configurations for STin2, rs25531 and 5-HTTLPR as well as for STin2 and 5-HTTLPR. Linkage Disequilibrium (LD) was calculated using HaploView Version 4.2.

Sensitivity analyses were conducted with G*Power 3.1.9 [42].

Bonferroni correction was conducted by dividing α by 6 (HA and neuroticism scales and three polymorphisms) leading to an adjusted level of significance of α = 0.008. Only MANCOVA results matching this criterion were presented in the results section but for reasons of transparency Table 3 also includes results below p = .05.

## Results

### Genetic analyses

The genotype frequencies for 5-HTTLPR (biallelic approach: LL: n = 851, SL: n = 1201, SS: n = 470; test for HWE: χ^2^ = 1.619, df=1, p = .203); triallelic approach (5-HTTLPR including rs25531: n = 677 (L’L’), n = 1226 (S’L’), and n = 619 (S’S’); test for HWE: χ^2^ = 1.872, df= 1, p = .171) as well as for STin2 VNTR (12/12: n = 1009, 12/10: n = 1107, 10/10: n = 345, 12/9: n = 45, 10/9: n = 16; test for HWE: χ^2^ =5.345, df=3, p = .064) were in Hardy-Weinberg equilibrium in the total sample of healthy participants as well as for inpatients (5-HTTLPR: biallelic approach: χ^2^ =1.448, df=1, p = .228; triallelic approach: χ^2^ = 0.479, df=1, p = .489; STin2: χ^2^ =0.467, df=3, p = .926; χ^2^ =0.479, df=1, p = .488).

There were no sex differences in genotype distributions between healthy controls (5-HTTLPR: biallelic approach: χ^2^ = 1.318, df=2, p = .517; triallelic approach: χ^2^ = 2.380, df=2, p = .304; STin2: χ^2^ = 1.162, df=4, p = .884) and inpatients (5-HTTLPR: biallelic approach: χ^2^ = 1.178, df=2, p = .555; triallelic approach: χ^2^ = .459, df=2, p = .795; STin2: χ^2^ = 1.596, df=4, p = .810).

Results of linkage analyses from our overall sample support previous findings [32], [43], [44], [45] showing that 5-HTTLPR and rs25531 were in strong LD, while there was only relative weak linkage disequilibrium between these two and STin2 (LD between each of two markers: HTTLPR x rs25531: D’=0.97, r^2^ = .042; HTTLPR x STin2 D’=0.51, r^2^ = .115; rs25531 x STin2: D’=0.55, r^2^ = .011).

### Age and sex effects, critical life events

Middle-aged participants showed a negative correlation between age and HA (r = −.06, p = .04). In inpatients and students, neuroticism (inpatients: r = −.182, p < .001; students r = −.140, p < .001) and harm avoidance (inpatients: r = −.173, p < .001; students: r = −.144, p < .001) were significantly negatively correlated with age. Across all participants, age significantly correlated positively with neuroticism (r = .098, p < .001) but not with HA (r = .001, p = .953). This is because inpatients are older (31.88 (SD= 12.61) versus 47.96 (SD= 9.64); F_(1,2967)_= 96.00, p < .001) and scored significantly higher on HA (F_(1,2967)_= 10.29, p < .001) and neuroticism (F_(1,2967)_= 4.003, p < .001) than healthy controls.

An influence of sex on HA (students: F_(1,1343)_= 84.32, p < .001; middle-aged: F_(1,1175)_= 15.24, p < .001; inpatients: F_(1445)_= 3.22, p = .073), as well as on neuroticism (students: F_(1,1343)_= 82.52, p < .001; middle-aged: F_(1,1175)_= 33.25, p < .001; inpatients: F_(1445)_= 4.805, p = .029) could be detected, with female participants having higher scores than male participants. Therefore, age was entered as a covariate and sex as an additional independent factor for both personality scales in the ensuing MANCOVA-models. Descriptive statistics for age, sex, stressful life events (SLEs) as well as mean HA and neuroticism scores separated by group are provided in Table 1.

**Table 1:** Age, Harm Avoidance (HA) score, neuroticism score and the number of stressful life events (SLE) separated by sex and survey group. Note: Means and Standard Deviations are presented.

	Students		Middle-aged		Inpatients	
Sex	Men n = 371	Women n = 974	Men n = 442	Women n = 735	Men n = 154	Women n = 293
Age	27.17 ± 8.772	23.52 ± 7.267	41,34 ± 11,54	39.65 ± 11.77	48,53 ± 9.718	47.67 ± 9.595
HA	13.491 ± 7.978	17.807 ± 7.598	13.871 ± 7.305	15.505 ± 6.737	21.629 ± 6.764	22.833 ± 6.721
Neuroticism	2.567 ± 0.719	2.944 ± 0.663	2.649 ± 0.916	2.961 ± 0.888	3.633 ± 0.829	3.817 ± 0.853
SLE	5.29 ± 3.711	4.11 ± 3.133	5.22 ± 3.43	5.32 ± 3.396	6.87 ± 4.024	7.28 ± 4.077

SLE correlated significantly with age in healthy controls (middle-aged: r = .200, p < .001; students: r = .335, p < .001), but neither with neuroticism (middle-aged: r = .004, p > .05; students: r = .059, p > .05) nor HA (middle-aged: r = −.024, p > .05; students: r = −.038 p > .05). Inpatients showed no significant correlation between SLE and age (r = .062, p > .05) as well as between SLE and neuroticism (r = .021, p > .05) but between SLE and HA (r = −.105, p = .027) a significant but weak negative correlation emerged, which does not hold Bonferroni correction. Therefore, stressful life events were not considered as covariate in the ensuing analyses. Analysing SLEs x 5-HTTLPR genotype interaction on personality revealed no significant results, neither for the total sample nor for subgroups.

### Genetic effects on neuroticism & HA

We conducted MANCOVAs with sex and the genetic variants as fixed factors, age as covariate, and HA and neuroticism as dependent variables for each of the three samples separately and for the total sample, to look for main effects as well as interaction effects dependent on the different allelic combinations. See Table 2 for descriptive statistics for HA and neuroticism scores separated by 5-HTTLPR, the triallelic approach, STin2, and sex, respectively. Moreover, Table 3 provides detailed results of the following multivariate models whereas the text only reports adjusted p-values with p < =.008.

**Table 2:** Descriptive statistics for HA and neuroticism dimensions separated by 5-HTTLPR genotype and triallelic approach, STin2 grouped models, survey group and sex. Note: Means and Standard Deviations are presented.

	Students		Middle-aged		Inpatients	
Males	HA	neuroticism	HA	neuroticism	HA	neuroticism
5-HTTLPR						
S/S	14.887 (8.82)	2.579 (.69)	15.064 (7.63)	2.776 (.91)	25.480 (5.48)	3.900 (.76)
S/L	13.593 (8.04)	2.613 (.77)	13.990 (6.85)	2.673 (.95)	20.563 (6.42)	3.486 (.93)
L/L	12.578 (7.32)	2.499 (.66)	13.132 (7.66)	2.557 (.87)	21.276 (7.18)	3.698 (.68)
Triallelic						
S’/S’	14.298 (8.49)	2.579 (.70)	14.359 (7.30)	2.677 (.87)	23.288 (6.04)	3.750 (.81)
S’/L’	13.373 (7.94)	2.585 (.75)	14.117 (716)	2.683 (.96)	20.781 (6.22)	3.548 (.91)
L’/L’	12.972 (7.59)	2.531 (.68)	13.056 (7.54)	2.568 (.87)	21.156 (7.80)	3.678 (.69)
STin2						
10R+	13.682 (8.31)	2.564 (.72)	14.401 (7.43)	2.757 (.95)	21.141 (6.77)	3.571 (.82)
10R-	13.237 (7.53)	2.571 (.72)	13.023 (7.04)	2.476 (.83)	22.355 (6.73)	3.726 (.83)
12R+	13.457 (8.02)	2.567 (.72)	13.773 (7.14)	2.612 (.89)	22.085 (6.35)	3.670 (.84)
12R-	13.679 (7.77)	2.570 (.73)	14.418 (8.21)	2.858 (1.01)	19.280 (8.35)	3.440 (.74)
Females	HA	neuroticism	HA	neuroticism	HA	neuroticism
5-HTTLPR						
S/S	17.519 (7.11)	2.937 (.62)	15.599 (6.55)	3.042 (.87)	23.305 (6.12)	3.746 (.88)
S/L	17.975 (7.38)	2.974 (.66)	15.559 (6.94)	2.936 (.89)	22.963 (6.68)	3.840 (.84)
L/L	17.722 (8.22)	2.901 (.69)	15.384 (6.56)	2.954 (.89)	22.380 (7.13)	3.830 (.80)
Triallelic						
S’/S’	17.680 (6.92)	2.933 (.60)	15.758 (6.48)	3.028 (.85)	23.288 (6.09)	3.808 (.84)
S’/L’	17.477 (7.56)	2.952 (.68)	15.641 (6.83)	2.966 (.89)	22.909 (6.80)	3.846 (.84)
L’/L’	18.573 (8.25)	2.939 (.69)	15.021 (6.78)	2.892 (.90)	22.260 (7.18)	3.773 (.89)
STin2						
10R+	17.884 (8.09)	2.932 (.66)	15.566 (6.82)	2.966 (.88)	22.732 (6.50)	3.841 (.82)
10R-	17.702 (6.88)	2.960 (.66)	15.421 (6.61)	2.955 (.90)	23.000 (7.10)	3.777 (.90)
12R+	17.719 (7.43)	2.945 (.66)	15.462 (6.73)	2.931 (.89)	22.960 (6.86)	3.815 (.85)
12R-	18.310 (8.53)	2.935 (.67)	15.796 (5.88)	3.172 (.84)	22.114 (5.89)	3.829 (.86)
Overall	HA	neuroticism	HA	neuroticism	HA	neuroticism
5-HTTLPR						
S/S	16.800 (7.69)	2.839 (.66)	15.400 (6.96)	2.943 (.89)	23.952 (5.99)	3.792 (.85)
S/L	16.817 (7.80)	2.879 (.71)	14.975 (6.94)	2.838 (.92)	22.132 (6.68)	3.717 (.88)
L/L	16.205 (8.30)	2.783 (.70)	14.525 (7.08)	2.802 (.91)	21.975 (7.15)	3.782 (.80)
Triallelic						
S’/S’	16.740 (7.54)	2.835 (.65)	15.246 (6.81)	2.900 (.88)	23.505 (6.05)	3.789 (.83)
S’/L’	16.412 (7.86)	2.856 (.72)	15.077 (6.98)	2.861 (.93)	22.190 (6.67)	3.745 (.88)
L’/L’	16.874 (8.45)	2.815 (.71)	14.249 (7.14)	2.765 (.91)	21.853 (7.40)	3.738 (.82)
STin2						
10R+	16.736 (8.36)	2.831 (.70)	15.111 (7.08)	2.885 (.91)	22.200 (6.62)	3.751 (.83)
10R-	16.455 (7.34)	2.851 (.70)	14.570 (6.85)	2.785 (.91)	22.767 (6.96)	3.759 (.88)
12R+	16.545 (7.83)	2.841 (.70)	14.840 (6.99)	2.813 (.90)	22.661 (6.69)	3.766 (.85)
12R-	17.020 (8.56)	2.833 (.70)	15.219 (6.96)	3.041 (.92)	21.087 (6.96)	3.688 (.84)

**Table 3:** Significant main and interaction effects for the genetic variants separated by group and NEM dimension (HA: Harm Avoidance; N: neuroticism). P-values obtained from MANCOVA with age as covariate and sex as additional fixed factor. Bold p-values reaches the Bonferroni adjusted alpha = .008. Of note, only gene variants and interactions with p ≤ .05 are listed.

	Students		Middle-aged		Inpatients		Overall	
NEM dimension	HA	N	HA	N	HA	N	HA	N
Genetic variants	*p-value*							
5-HTTLPR	-	-	-	-	**.005**	-	.018	-
5-HTTLPR * gender	-	-	-	-	.026	.043	-	-
triallelic	-	-	-	-	.045	-	-	-
10R+	-	-	-	**.008**	-	-	-	-
10R+* gender	-	-	-	.014	-	-	-	-
12R+	-	-	-	**.002**	.047	-	-	-
L+	-	-	-	-	**.001**	-	.012	-
L+* gender	-	-	-	-	.016	.05	.021	-
S+	-	.045	-	-	-	-	.037	-
triallelicL+	-	-	-	-	.014	-	-	-
S+* 10R+	-	-	-	.041	-	-	-	-
S+* 10R+* gender	-	-	-	.027	-	-	-	-
S+* 12R+	-	-	-	-	.032	-	-	-
triallelicL+* 10R+	-	-	-	-	-	-	.027	-
triallelicL+* 12R+	.045	-	-	-	-	-	-	-
triallelicL+* 12R+*gender	-	-	-	-	-	-	.036	-
triallelicS+* 10R+*gender	-	-	-	.046	-	-	-	-
10/LA * gender	-	-	-	.047	-	-	-	-
12/LA	-	-	**.00017**	**.000006**	-	-	**.001**	**.002**
12/LA * gender	-	-	-	.046	-	-	.017	-
10/SA	-	-	-	**.00017**	-	-	-	**.005**
12/LG	**.002**	.023	-	-	-	-	**.005**	-
10/L * gender	-	-	-	.032	-	-	-	-
12/L	.035	-	**.00017**	**.000005**	-	-	**.00003**	**.00044**
12/L * gender	-	-	.031	.018	-	-	**.006**	-
10/S	-	-	-	**.000016**	-	-	-	**.002**

#### 5-HTTLPR

With respect to 5-HTTLPR genotype there was only a significant main effect for HA (F_(2440)_= 5.375, p = .005, η^2^ = .024) in the inpatient group, with highest scores in homozygous S allele carriers followed by heterozygote S/L carriers. Post hoc analyses showed that carriers of the S/S genotype differed significantly from the S/L (p = .002) and the L/L group (p = .004) but there was no difference in HA between the latter two groups. Accordingly, in the biallelic approach, L+ carriers have significant lower HA scores than homozygous S-allele carriers (F_(1440)_= 10.594, p = .001, η^2^ = .023). Neither in one of the three groups nor in the total sample there was a main effect of 5-HTTLPR on neuroticism.

Of note, using the triallelic genotype that includes rs25531, no significant main effects on HA or neuroticism were observed.

#### STin2

The STin2.10R model revealed a main effect on neuroticism in the middle-aged sample (F_(1,1172)_= 7.116, p = .008, η^2^ = .006) with carriers of the 12/12 (and 12/9) genotype having lower neuroticism scores (mean 2.780 (.91) versus 2.885 (.91)).

The model with STin2.12R also showed a significant effect on the neuroticism dimension in the middle-aged sample (F_(1,1172)_= 9.790, p = .002, η^2^ = .008) with carriers of at least one 12R allele having lower neuroticism scores (mean 2.813 (.91) versus 3.041 (.93)).

#### 5-HTTLPR x STin2

We calculated MANCOVA models for all possible combinations of independent variables. None of these interaction effects showed a significant effect on HA or on neuroticism. A detailed overview of these findings is presented in Table 3.

### Haplotype analyses

In analogy with previous findings in the literature we performed additional haplotype analyses. This strategy allows to consider the individual chromosomal genotype and thereby other functional variants which are in LD with those under investigation.

For the three polymorphic loci 10 haplotypes resulting in 31 different haplotype combinations were observed in the total sample. The most frequent haplotypes that constituted 98.2% of all haplotypes were 12/SA (36%), followed by 10/LA (29.6%), 12/LA (21.3%), 10/SA (6.2%) and 12/LG (5.1%). See Table 4 for descriptive statistics for HA and neuroticism scores separated by haplotype and sex.

**Table 4:** Descriptive statistics for HA and neuroticism dimensions separated by haplotype, survey group and sex. Values are presented as mean (SD).

	Students		Middle-aged		Inpatients	
Males	HA	neuroticism	HA	neuroticism	HA	neuroticism
12/SA	13.930 (8.35)	2.582 (.73)	14.060 (6.99)	2.651 (.92)	22.416 (6.25)	3.624 (.91)
10/LA	13.623 (8.13)	2.578 (.73)	14.367 (7.55)	2.741 (.96)	20.988 (6.76)	3.530 (.84)
12/LA	12.625 (7.27)	2.540 (.74)	12.462 (6.95)	2.428 (.81)	21.214 (7.17)	3.768 (.77)
12/LG	11.650 (6.47)	2.488 (.64)	12.844 (6.18)	2.522 (.80)	20.200 (5.40)	3.567 (.88)
10/SA	14.047 (8.97)	2.600 (.78)	15.672 (7.28)	3.034 (.95)	19.154 (7.72)	3.654 (.85)
12/S	13.893 (8.32)	2.588 (.74)	14.102 (6.91)	2.653 (.91)	22.416 (6.25)	3.624 (.91)
10/L	13.639 (8.10)	2.588 (.74)	14.371 (7.46)	2.735 (.95)	21.035 (6.73)	3.535 (.83)
12/L	12.470 (7.10)	2.523 (.71)	12.510 (6.81)	2.446 (.80)	20.956 (6.86)	3.743 (.78)
10/S	14.057 (9.32)	2.549 (.75)	15.687 (7.69)	3.078 (.97)	19.154 (7.72)	3.654 (.85)
Females	HA	neuroticism	HA	neuroticism	HA	neuroticism
12/SA	17.730 (7.15)	2.965 (.65)	15.599 (6.88)	2.954 (.89)	23.169 (6.66)	3.777 (.87)
10/LA	17.990 (8.17)	2.933 (.67)	15.694 (6.83)	2.953 (.88)	22.500 (6.69)	3.831 (.84)
12/LA	18.102 (7.62)	2.971 (.71)	14.896 (6.87)	2.874 (.91)	22.745 (7.59)	3.842 (.89)
12/LG	15.533 (7.12)	2.762 (.60)	15.705 (5.93)	2.966 (.82)	24.417 (5.76)	4.062 (.70)
10/SA	17.694 (7.98)	2.931 (.61)	15.179 (6.57)	3.128 (.89)	23.333 (5.08)	4.030 (.66)
12/S	17.698 (7.13)	2.962 (.65)	15.523 (6.88)	2.945 (.88)	23.150 (6.61)	3.775 (.86)
10/L	17.970 (8.11)	2.933 (.67)	15.603 (6.86)	2.942 (.88)	22.500 (6.61)	3.832 (.84)
12/L	17.773 (7.62)	2.936 (.70)	15.135 (6.64)	2.896 (.89)	23.053 (7.42)	3.886 (.87)
10/S	17.875 (8.16)	2.941 (.62)	15.614 (6.43)	3.207 (.88)	23.467 (5.29)	4.067 (.65)
overall	HA	neuroticism	HA	neuroticism	HA	neuroticism
12/SA	16.723 (7.67)	2.864 (.70)	15.044 (6.95)	2.845 (.91)	22.917 (6.52)	3.726 (.88)
10/LA	16.779 (8.39)	2.835 (.70)	15.177 (7.14)	2.870 (.92)	21.980 (6.74)	3.727 (.85)
12/LA	16.606 (7.91)	2.853 (.74)	13.962 (6.99)	2.703 (.90)	22.188 (7.45)	3.815 (.85)
12/LG	14.338 (7.13)	2.677 (.62)	14.737 (6.14)	2.816 (.84)	22.795 (5.93)	3.872 (.80)
10/SA	16.754 (8.37)	2.846 (.67)	15.390 (6.86)	3.088 (.91)	22.152 (6.15)	3.924 (.73)
12/S	16.675 (7.65)	2.862 (.70)	15.009 (6.92)	2.840 (.91)	22.907 (6.49)	3.725 (.88)
10/L	16.762 (8.33)	2.837 (.70)	15.120 (7.12)	2.861 (.91)	22.000 (6.67)	3.731 (.85)
12/L	16.271 (7.84)	2.819 (.72)	14.138 (6.82)	2.725 (.88)	22.269 (7.27)	3.832 (.84)
10/S	16.966 (8.57)	2.847 (.67)	15.645 (6.96)	3.153 (.92)	22.163 (6.35)	3.942 (.73)

For the haplotype 12/LA significant main effects on both NEM dimensions could be detected in the total sample. For neuroticism (F_(1,2964)_= 9.225, p = .002, η^2^ = .003) as well as HA (F_(1,2964)_= 11.019, p = .001, η^2^ = .004) carriers of the 12/LA haplotype had lower NEM scores than non-carriers. Also in the middle-aged subsample both main effects became significant (neuroticism: F_(1,1172)_= 20.594, p < .001, η^2^ = .017; HA: F_(1,1172)_= 14.215, p < .001, η^2^ = .012).

Regarding the haplotype 12/LG only the main effect on HA became significant in the total sample (F_(1,2964)_= 7.808, p = .005, η^2^ = .003) as well as in the students subgroup (F_(1,1340)_= 9.778, p = .002, η^2^ = .007). 12/LG carriers are associated with lower HA score than non-carriers.

The haplotype 10/SA shows only a significant main effect on neuroticism in the total sample (F_(1,2964)_= 7.796, p = .005, η^2^ = .003) and in the middle-aged (F_(1,1172)_= 14.245, p < .001, η^2^ = .012). Carriers of at least one 10/SA haplotype are associated with higher neuroticism scores than non-carriers.

Excluding rs25531 results in 6 haplotype configurations with 12/S (36.7%), 10/L (30.6%), 12/L (25.7%), 10/S (5.6%), 9/L (1.3%) and 9/S (0.1%), respectively and 15 different haplotype pair combinations in the total sample.

**Fig. 1:**
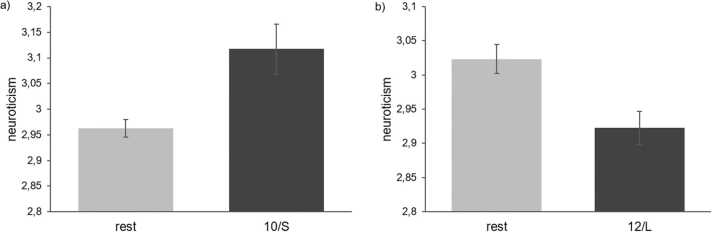
Mean ( ± SEM) neuroticism scores for haplotype 10/S (a) as well as 12/L (b) against all other haplotype configurations (rest) depicted for the total sample (n = 2969).

The haplotype constituting STin2.12 and 5-HTTLPR L allelic variation mirrors strong main effects on both NEM dimensions (HA: F_(1,2964)_= 17.448, p < .001, η^2^ = .006; neuroticism: F_(1,2964)_= 12.358, p < .001, η^2^ = .004; Fig. 1) with carriers of at least one 12/L allele having lower scores than non-carriers as well as men having significantly lower HA scores than women (F_(1,2964)_= 7.678, p = .006, η^2^ = .003). Looking at the subsamples revealed that the effect is mainly driven by the middle-aged group (HA: F_(1,1172)_= 14.196, p < .001, η^2^ = .012; neuroticism: F_(1,1172)_= 20.939, p < .001, η^2^ = .018). For both personality dimensions lower scores are characterized by the 12/L haplotype and male sex.

The haplotype configuration 10/S mirrors the effects of 10/SA haplotype leading to a significant main effect on neuroticism dimension in the total sample (F_(1,2964)_= 9.948, p = .002, η^2^ = .003; Fig. 1) with carriers of at least one 10/S haplotype having higher neuroticism scores. Again, in the subsamples the effect is driven by the middle-aged (F_(1,1172)_= 18.756, p < 001, η^2^ = .016).

## Discussion

The literature is full of studies investigating a possible link between the 5-HTT gene and traits of negative emotionality (NEM) with heterogeneity in results in part based on methodological issues, e.g., differences in assessment tools (HA vs. N), low sample sizes in the primary studies and inclusion of rs25531 or not. The present study overcomes these shortcomings by investigating a possible interaction as well as haplotype effects on both NEM dimensions regarding STin2 and (triallelic) 5-HTTLPR VNTRs. Moreover, our study was conducted in a large sample of healthy young and middle-aged Caucasians as well as inpatients suffering from burnout/stress-related pathology. Results revealed a significant main effect of 5-HTTLPR on HA in inpatients explaining 2.4% of variance with L allele carriers having lower scores than homozygous S allele carriers replicating previous findings associating higher NEM values with the 5-HTTLPR S allele. For STin2 a significant association of the 12R allele with lower neuroticism scores could be detected in healthy middle-aged participants. Besides these significant main effects, no interaction effects could be observed as well as no effect of stressful life events on NEM expression occurred. However, according to existing literature a tendency of the L and 12R alleles for lower NEM values could be observed at least on the descriptive level across the different groups. Moreover, the haplotype approach resembles genotype effects in the total sample as well as for the group of middle-aged participants. Here, according to previous research [32] the 12/L haplotype goes along with significantly lower NEM scores. Moreover, the 10/S haplotype shows significant association with neuroticism in the middle-aged group with carriers of at least one 10/S haplotype having higher neuroticism scores. The inclusion of rs25531 in the haplotype analysis changes results slightly in the way that the 12/LG (LG = S) haplotype still becomes significant for HA in the total sample and additionally in the student subsample but no longer in the middle-aged group. Thus, the effects of the 5-HTTLPR L allele and the Stin2.12 became weaker in presence of the rs25531 G allele (compared to the A allele). This makes sense considering the idea that the LG allele is more similar to the 5-HTTLPR S allele than the L allele. In line with this, the significant association of 12/L with both NEM dimensions was mirrored nearly identical by the 12/LA haplotype. Further, it seems that in our sample the presence of the 12R allele in the 12/LG haplotype predispose to lower HA values in younger participants. In sum, the additional examination of rs25531 seems to be favourable when analysing HA and neuroticism on the haplotype level at least in healthy subjects. Moreover, the analysis of haplotypes of HTTLPR (and rs25531) and STin2 provides stronger evidence for an association of these genetic variants with NEM in comparison to the consideration of single VNTRs in isolation. Nevertheless, further examination of the haplotypes including these three polymorphisms and inclusion of additional SNPs, which are in LD and may additionally strengthen the association, is necessary. This is promising because the chromosomal genotype resembles (much stronger than variants solely) the 3D genomic organization as well as interaction of non-coding regulatory elements taking place in vivo and most likely influencing the expression of the 5-HTT gene [29], [31].

Especially for the 5-HTTLPR VNTR located in the promoter region and the STin2 VNTR having allele-specific enhancer-like properties [17], a joint effect on transcriptional regulation further modulated by the repeat copy number within the VNTR can be assumed. In general, the 3D folding of non-coding regions which allows long-distance interactions of promoter and enhancer regions is crucial for efficiently initiating transcription [29]. Accordingly, studies investigating the interplay of both VNTRs by transactivation assays using reporter gene plasmids showing combinatorial effects of the VNTR polymorphisms are promising [31]. Obviously, in vivo the 3D structure of the chromatin leading to a promoter-enhancer loop is much more complex and it depends e.g., on tissue-specific transcription factors as well as epigenetic modifications which further modulates the interaction of regulatory elements thereby influencing the transcription activation process regulating the 5-HTT expression level.

Epigenetic modifications occur throughout developmental stages and our whole live and can be triggered by critical live events. If these modifications occur in regulatory regions, they can disturb the tight spatio-temporal regulation of a gene leading to altered gene expression which modulates neurodevelopment leading to distinct differences in personality traits or could result in a genetic disorder [46]. Therefore, a major modulator of mental wellbeing is how our genes are regulated in response to (critical or stressful) life experiences [30]. In our study stressful life events experienced across the life span were measured by a self-report questionnaire. No significant association with personality traits was observed neither for healthy participants nor inpatients with stress-reactive phenotypes like depression and/or burnout. The amount of experienced SLEs does not differ between 5-HTTLPR genotype, so the genetic makeup does not predispose to the experience of more SLEs. Moreover, in line with recent meta-analyses which do not support the interaction hypothesis between 5-HTTLPR genotype and stress on depression [47] as well as previous ones including neuroticism [48], the current study does not provide evidence for a 5-HTTLPR genotype x SLE interaction on HA or neuroticism scores neither in the total sample nor in the subgroups of healthy participants or inpatients. Despite this, a study with monozygotic twins highlights the relevance of analysing 5-HTT promoter DNA methylation in the context of neural responses to negative stimuli. Independent of DNA sequence effects, twins with higher peripheral promoter methylation levels showed greater frontal-limbic brain responses to fearful and sad facial expressions, relative to the co-twins with lower SLC6A4 methylation [27] supporting that environmental factors (e.g. SLEs) may influence aspects of the serotonin system and thereby emotional processing via changes of 5-HTT promoter methylation.

To date, this is to the best of our knowledge the largest study to examine combinatorial effects of the serotonergic variants 5-HTTLPR, rs25531 and STin2 regarding personality traits. In general, the significant findings had small effect sizes (i.e., explained 2.4% of variance) with respect to the significant main effect of 5-HTTLPR S allele on HA scores in inpatients. However, no significant interaction effect could be observed neither in the three subgroups nor in the total sample. This has to be evaluated in the light of the sample size. For inpatients, a sensitivity analysis based on an alpha value of.05 and a sample size of n = 447, resulted in an effect size eta^2^ of.028. This means that the presence of an interaction effect of smaller size cannot be excluded with sufficient certainty. Accordingly, the students’ sample had an eta^2^ of.010 and the middle-aged sample had an eta^2^ of.011. All in all, the available data do not allow to exclude the presence of an interaction effect of.010 or smaller. Moreover, our sample comprises healthy young as well as middle aged Caucasians and in the total sample also inpatients were included in the analyses. With this we covered a wide range in age as well as in NEM scores thereby increasing the statistical power and increasing the representation of the continuum model of personality. Of course, cautious interpretation due to unknown sources of population stratification affecting association results is warranted. Besides, haplotype effects have to be replicated in an independent sample and due to the low frequency of the rare STin2.9 allele and the resulting haplotypes, even larger sample sizes are needed to detect possible effects of this variant.

A further potential shortcoming of this study is that SLEs were measured with a self-report survey. As observed in previous research, more recent life events seem to have a stronger effect on mood and depression. Furthermore, it has been shown that particularly depressed patients are more likely to report life events due to mood congruence recall bias [47].

Moreover, the effect of only one gene (5-HTT) on personality traits was considered. Of course, serotonergic neurotransmission depends on many other genes and gene variants each with a very small effect. As raised by several other authors pathway analyses including genes of the same biological pathway are necessary. Besides, further studies on gene-gene interactions as well as further gene-environment interactions (e.g., maintaining good mental health via diet and exercise) should be considered. Future studies should include epigenetic as well as transcriptional analyses to rule out whether 5-HTT haplotypes are associated with expression level and to examine whether specific methylation or histone patterns as well as abundance of specific ncRNA further fine-tune 5-HTT protein occurrence. These analyses could highlight the influence of early lifetime as well as acute stressors on serotonin, respectively serotonin transporter availability in the synaptic cleft and consider a role of changing 5-HT levels in clinical affective disorders as well as in mood regulation in healthy individuals.

In conclusion, the investigation of the haplotypes of these three polymorphisms, as well as the inclusion of a number of additional SNPs of the 5-HTT gene in further large-scale studies, is promising to further elucidate their contribution to the genetic basis of NEM from the healthy to the pathological range.

## Declaration of Competing Interest

The authors declare that they have no known competing financial interests or personal relationships that could have appeared to influence the work reported in this paper.
